# American Dental Association

**Published:** 1892-05

**Authors:** 


### AMERICAN DENTAL ASSOCIATION.

Special attention is called to the notice of the Executive Committee of this Association on another page. This is a timely request, and it is hoped that all who contemplate writing papers will comply with it, and prepare their essays in duplicate, as desired. It would be better if four type-written copies were made, instead of two. This does not involve much extra trouble or expense, and would save much unnecessary anxiety on the part of the secretary.

It is well known that the leading journals all desire to make abstracts, and it is to the interest of the Association that its proceedings should have the widest circulation. To do this with one copy presented is impossible, and at previous meetings this has been a constant source of annoyance. Very few, it is imagined, are aware of the labor involved in preparing a report such as has appeared in the INTERNATIONAL DENTAL JOURNAL from month to month; and when it is considered that abstracts must be made and discussions reported during the sessions of the meeting, the request cannot seem an unreasonable one. It is hoped that the secretary of each section will enforce this by a special notice.

